# Prevalence of malaria parasites among blood donors in two hospitals in Enugu metropolis, Nigeria

**DOI:** 10.1016/j.htct.2025.103858

**Published:** 2025-06-18

**Authors:** Samuel ThankGod Aluh, Patience Obiageli Ubachukwu, Kyrian Ikenna Onah, Gabriel Adebayo Oladepo, Chidi Ole Ukwen, Fupsin Rimamkirnde

**Affiliations:** a Parasitology and Public Health Unit, Department of Zoology and Environmental Biology, University of Nigeria, Nsukka, Nigeria; b Ecology Unit, Department of Zoology and Environmental Biology, University of Nigeria, Nsukka, Nigeria

**Keywords:** Blood transfusion, Malaria, WHO, Blood donor

## Abstract

**Introduction:**

Screening of blood donors for malaria parasites as recommended by the World Health Organization (WHO) is currently not included in the protocols and procedures for pre-screening blood donors of many private and public health facilities in Nigeria.

**Methods:**

A cross-sectional study was conducted of voluntary, family, and remunerated blood donors in two hospitals in the Enugu metropolis. A well-structured questionnaire was used to collect demographics and blood donation history data. Five milliliters of blood were collected from each blood donor, of which 2 mL were used to screen for malaria parasites.

**Results:**

Three hundred and seventy-seven blood donors participated in the study with 148 (39.3 %) being malaria-positive. Most of the blood donors were in the age groups 16–25 and 26–35 years old with prevalences of 40.0 % and 44.1 %, respectively. The prevalence of malaria in both age groups was high compared to the 36–45 years age group (26.7 %). Still, the overall difference in malaria prevalence across the four age groups was not statistically significant (χ^2^ = 5.437; *p*-value = 0.142). The majority (*n* = 290; 76.9 %) of the donors were male, while 87 (23.1 %) were female. Although female blood donors had a higher prevalence of malaria (47.1 %) compared to male donors (36.9 %), the difference was not statistically significant (*p*-value = 0.057).

**Conclusion:**

The high prevalence of malaria in the studied area, suggests the need for careful screening of blood samples of blood donors for malaria parasites.

## Introduction

Malaria has become one of the most widespread and most important single disease entities of the tropics with unprecedented high morbidity and mortality. In 2022, there were an estimated 249 million malaria cases reported across 85 malaria-endemic countries and regions. This marked an increase of 5 million cases compared to 2021. The primary contributors to this rise were Pakistan (+2.1 million), Ethiopia (+1.3 million), Nigeria (+1.3 million), Uganda (+597,000), and Papua New Guinea (+ 423,000). In comparison, in 2015, the baseline year for the Global Technical Strategy for Malaria 2016–2030, there were approximately 231 million malaria cases. Malaria case incidence decreased from 81 per 1,000 people at risk in 2,000 to 57 per 1,000 in 2019. Despite a slight 3 % increase in 2020, the incidence rates have remained stable over the past three years, with the incidence rate in 2022 recorded at 58 per 1,000 people at risk.^1^ Studies have shown that there may be about 350–500 million cases of malaria each year. ^2^^,^^3^ About 41 % of the world population is said to be at risk of infection causing up to 2.7 million deaths annually.^3^

There are high levels of blood-demanding health conditions in Nigeria such as severe anemia in pregnancy and some hemorrhagic complications that occur particularly during childbearing, and after road traffic accidents, hence the need for blood transfusions. These amplify the possibility of the transmission of blood-borne diseases.^4^ The spread of multidrug-resistant *Plasmodium falciparum* can be acquired equally by transfusion and the infection can be life-threatening in a patient population that is impoverished and vulnerable.^5^^,^^6^ The frequent occurrence of blood-transfused malaria has raised concerns about potential infection worries in the fight to eradicate malaria. Also, children under the age of 5 years old, pregnant women, accident victims, immune-suppressed patients, and others requiring emergency blood transfusions are constantly at risk of its attendant infections.^7^

In Sub-Saharan Africa, where malaria is endemic, the screening of blood donors for malaria parasites is often not prioritized. This occurs despite international policies mandating that donated blood be quality-assured and free from transfusion-transmissible infections, including malaria.^8^ As most of the people living in malaria-endemic regions are semi-immune, the test used to screen blood must be sensitive. This research sought to assess the prevalence of malaria parasites among blood donors in two hospitals in Enugu Metropolis, Nigeria.

## Methods

### Population and design

This was a cross-sectional study. The sample for this study included 377 individuals (remunerated, volunteer, and family members) who donated blood at two hospitals, the blood donor units of the University of Nigeria Teaching Hospital (UNTH) and the Enugu State University Teaching Hospital (ESUTH) in the Enugu metropolis, Nigeria. The sample size was determined using the standard formula of Cochran for calculating the minimum sample size.^9^ A semi-structured interviewer-administered questionnaire was administered to all consenting participants to obtain information on their sociodemographic status. Ethical clearance for this study was obtained from the Enugu State Ministry of Health and Enugu State University Teaching Hospital, Parklane Research Ethics Committee.

### Laboratory procedure for blood sample collection and malaria screening

From each donor, 5 mL of whole venous blood was collected for preliminary screening to assess donor suitability. Of this volume, 2 mL was subsequently aliquoted into ethylenediaminetetraacetic acid (EDTA) bottles. Malaria was diagnosed microscopically by staining thick and thin blood films on grease-free glass slides to visualize malaria parasites using Giemsa staining. Thick and thin blood films were prepared according to the technique outlined by the World Health Organization.^3^

### Statistical analysis

The data were analyzed using the Statistical Package for Social Sciences (SPSS) version 23.0 (IBM Corporation, Armonk, USA). The prevalence of malaria was estimated by the chi-square test. The questionnaire responses are summarized as frequency distributions. Statistical significance was determined at an alpha level of 0.05 (*p*-value <0.05), corresponding to a 95 % confidence level.

## Results

### Blood donor demographic characteristics

The demography of blood donors who partook in the study is outlined in Table 1. All 377 individuals tested for malaria answered the study questionnaire. The blood donors were aged between 16 and 65 years. Most of the blood donors were in the 16- to 25- and 26- to 35-year-old age groups with prevalences of malaria of 40.0 % and 44.1 %, respectively. There were more than three times more male blood donors than female blood donors (76.9 % versus 23.1 %) yet female blood donors had a higher prevalence of malaria. Moreover, there were over three times more unmarried blood donors than their married counterparts (76.7 % versus 23.3 %). Although the prevalences of malaria in both the younger age groups were higher than the 36- to 45-year-old age group (26.7 %), the overall difference in prevalence across the four age groups was not statistically significant (χ^2^ = 5.437; *p*-value = 0.142).

**Table 1:** Demographic characteristics and prevalence of malaria infection among blood donors in two hospitals in Enugu metropolis, Nigeria.

Variable		*n*	Frequency (%)	Infected (%)	
Age (years)	16–25	200	53.1	80 (40.0)	χ^2^ = 5.437; df = 3 *p*-value = 0.142
	26–35	111	29.4	49 (44.1)	
	36–45	60	15.9	16 (26.7)	
	46–65	6	1.6	3 (50.0)	
Gender	Male	290	76.9	107 (36.9)	χ^2^ = 2.937; df = 1 *p*-value = 0.057
	Female	87	23.1	41 (47.1)	
Marital status	Single	289	76.7	108 (37.4)	χ^2^ = 1.849; df = 1 *p*-value = 0.109
	Married	88	23.3	40 (45.5)	
Level of education	Primary	15	4.0	8 (53.3)	χ^2^ = 3.371; df = 2 *p*-value = 0.185
	Secondary	162	43.0	56 (34.6)	
	tertiary	200	53.1	84 (42.0)	
Occupation	Civil servant	53	14.1	32 (60.4)	χ^2^ = 20.104; df = 5 *p*-value = 0.001
	Business	134	35.5	49 (36.6)	
	Professionals	20	5.3	8 (40.0)	
	Skilled artisans	27	7.2	7 (25.9)	
	Students	131	34.7	52 (39.7)	
	Unemployed	12	3.2	0 (0)	

df: degree of freedom.

### Blood donation history

The blood donors fell into three categories: those who had to donate because family members needed blood (*n* = 277; 73.5 %), those making voluntary donations (*n* = 81; 21.5 %) and those donating for remuneration (*n* = 19; 5.0 %). Most (*n* = 241; 63.9 %) of the blood donors are repeated donors compared to 136 (36.1 %) doing so for the first time. The majority of the repeat donors had donated blood about six months previously (*n* = 138; 57.3 % - Table 2).

**Table 2:** Blood donation history.

Blood donor attribute	*n* (%)
When was the last time you donated blood? (*n* = 241)	
1–4 weeks ago	57 (23.7)
6 months ago	138 (57.3)
I cannot remember	46 (19.1)
Were you tested for malaria before donating blood?	
Yes	3 (0.8)
No No response	244 (64.7) 130 (34.5)
Where was the test taken? (*n* = 3)	
Hospital	2 (66.7)
Laboratory	1 (33.3)
Source of knowledge about blood donation	
Television	20 (5.3)
Internet	28 (7.4)
Newspaper	2 (0.5)
People	297 (78.8)
Books	18 (4.8)
Others	12 (3.2)

### Prevalence of malaria parasites among blood donors

Blood group *O*+ was the dominant blood type registered in this study followed by *A*+, *B*+, O-, and B-. No blood group A- or AB individuals were registered in this study. Of the 188 (*O*+), 32 (O-), 88 (*A*+), 53 (*B*+) and 16 (B-) blood group subjects examined, the malaria infection rates were 43.1 %, 34.4 %, 25.0 %, 58.5 % and 18.8 %, respectively. The chi-square analysis (χ^2^ = 20.020; *p*-value = 0.000) revealed a significant association between the blood group and infection (Table 3).

**Table 3:** Prevalence of malaria parasites grouped by blood group and donation history.

**Variable**	***n***	**Infected *n* (%)**	
Frequency of blood donation			
First-time donors	136	59 (43.4)	χ^2^ = 1.116; df = 1 *p*-value = 0.172
Repeat donors	241	49 (20.3)	
Type of blood donor			
Voluntary	81	37 (45.7)	χ^2^ = 1.788; df = 2 *p*-value = 0.409
Family	277	104 (37.5)	
Remunerated	19	7 (36.8)	
Blood group			
*A*+	88	22 (25.0)	χ^2^ = 20.020; df = 4 *p*-value = 0.000
*B*+	53	31 (58.5)	
B-	16	3 (18.8)	
*O*+	188	81 (43.1)	
O-	32	11 (34.4)	

df: degree of freedom.

## Discussion

The prevalence of malaria parasites among blood donors in two hospitals in Enugu Metropolis was studied. The result of this study revealed that of 377 blood donors screened for malaria parasites, 148 (39.3 %) were malaria-positive. This high prevalence of malaria parasites was similar to prevalences reported in different studies of blood donors in Southeastern Nigeria (40.9 %, 30.2 %, and 51.5 %).^4^^,^^7^^,^^10^ The implication of this for blood transfusions is enormous; blood transfusions carry the risk of transmitting malaria to hospitalized patients who are in dire need of blood. The risk of the recipient developing transfusion-transmitted malaria (TTM) is however dependent on other factors including the immune status and parasite density. Malaria prevalence rates in blood donors across Africa vary widely and depend on the local endemicity and transmission season. This study is contrary to a study conducted among blood donors in a hospital in Kumasi, Ghana where a low prevalence of malaria was recorded^11^ and a study in Sudan also reported a figure lower than the current study (13 %).^12^

The recorded parasite density in blood films (“+” and “++”) was not considered indicative of severe infection. This observation was cited by a laboratory scientist at one of the study hospitals as a rationale for excluding malaria testing from the routine pre-screening protocol for assessing blood donor eligibility. However, preservation of blood at 4 °C does not destroy the parasites, and even one to two parasites per mL of blood, which may be undetected on thick or thin blood films, are sufficient to transmit the illness. ^13^

The majority of blood donors in this study were males (*n* = 290; 76.9 %). The reason for the low number of female blood donors in this study (*n* = 87; 23.1 %) could be because of physiological issues such as menstruation, pregnancy, lactation, and iron deficiency anemia, which hinder them from donating blood regularly. Furthermore, the percentage of infections in this study was significantly higher in females than in males (47.1 % versus 36.9 %). This finding concurs with previous studies that reported a higher infection rate in females than in males.^14^, ^15^, ^16^, ^17^, ^18^ Another reason for the higher prevalence among females may be the contemplation of differences in disease susceptibility or reporting. However, there appears to be no scientific evidence linking malaria to gender.^18^ Generally, males donate blood more often than females, particularly in developing countries.^19^ The reason has been attributed to socio-cultural influences and beliefs.^20^

This study shows that the 46- to 65-year-old age group had the highest percentage of malaria infection. This finding did not agree with the observations in some previous studies where higher prevalences among younger age groups were recorded.^4^^,^^13^^,^^21^ The different observations in this study may be a result of the level of exposure, number of patients examined, low immunity, and lifestyle. Prevalence of malaria among 16- to 25- and 26- to 35-year age groups was also high compared to the 26.7 % in the 36- to 45-year age group, nonetheless the overall difference in prevalence across the four age groups was not statistically significant (χ^2^ = 5.437; *p*-value = 0.142).

From this present study, those identified as being married had the highest number of positive cases for malaria (*n* = 40; 45.5 %) compared to unmarried blood donors (*n* = 108; 37.4 %). This conforms with a previous study conducted in a general hospital in Minna, Niger State, Nigeria.^22^ The high malaria prevalence among married women was attributed to the existence of stagnant water around the homes which provides an environment for mosquitoes to breed, while another study with a high malaria prevalence among blood donors in Kaduna, Nigeria could not attribute the high rate to any specific reason.^23^

All blood groups were found to be parasitized with the malaria parasite in this present study indicating that no blood group can be exempted. Therefore, malaria can be transmitted through the transfusion of any of the different blood groups. Blood group *B*+ donors had the highest prevalence (58.5 %). The finding in this study is similar to a previous study in Southeastern Nigeria,^24^ but does not agree with other studies that recorded a higher prevalence of malaria among blood group *O*+ individuals.^25^^,^^26^

First-time donors had a higher prevalence (*n* = 59; 43.4 %) compared to repeat donors (*n* = 49; 20.3 %). The high percentage of malaria among new blood donors as seen in this study could be that previous donors might have had antimalaria treatment or were not exposed to malaria parasites before donating blood.

Those who identified themselves as voluntary blood donors had the highest prevalence of infection (*n* = 37; 45.7 %) while family or replacement blood donors and remunerated donors had prevalences of 37.5 % and 36.8 %, respectively. This present study contradicts previous findings where all studies recorded higher malaria infection rates in family blood donors.^23^^,^^27^

The highest infection rate was observed in donors with primary education (53.3 %), followed by those with tertiary education (42.0 %) while the lowest rate was in donors with secondary education (34.6 %). In contrast to a previous study in the Abagana community in Eastern Nigeria, the lowest prevalence of malaria was observed in individuals with tertiary education.^28^ One observation argued that those with tertiary education have better knowledge about the mode of transmission of malaria and ways to prevent and control the disease, thus they protect themselves better.^29^ The highest prevalence observed in the primary education group is also in agreement with the suggestion that those with little or no education may not have good knowledge about transmission and control of the disease.

## Conclusion

The prevalence of infection by the malaria parasite in donors, as seen in this present study, is significantly high. In as much as blood transfusion is required in severe anemia due to malaria and other illnesses, safe blood transfusion should be guaranteed. As the world searches for ways to curtail the spread and mortality due to malaria, there is an urgent need to screen blood donors for malaria parasites before donation. This is a neglected but important public health issue in Nigeria where malaria is endemic.

## Author contributions

ST: Conceptualization, data curation, original draft; POU: Supervision, review and editing; KIO: Review; GAO: Review; COU: Review; FR: Review.

## Funding

There was no funding for this research project.

## Conflicts of interest

The authors declare no conflicts of interest.
